# COVID-19 Hurricane: Recovering the Worldwide Health System with the RE.RE.RE. (REsponse–REstoration–REengineering) Approach—Who Will Get There First?

**DOI:** 10.3390/healthcare10040602

**Published:** 2022-03-23

**Authors:** Luigi Vetrugno, Cristian Deana, Salvatore Maurizio Maggiore

**Affiliations:** 1 Department of Medical, Oral and Biotechnological Sciences, University of Chieti-Pescara, 66100 Chieti, Italy; luigi.vetrugno@unich.it; 2 Department of Anesthesiology, Critical Care Medicine and Emergency, SS. Annunziata Hospital, 66100 Chieti, Italy; salvatore.maggiore@unich.it; 3 Department of Anesthesia and Intensive Care, Health Integrated Agency of Friuli Venezia Giulia, 33100 Udine, Italy; 4 Department of Innovative Technologies in Medicine and Dentistry, Gabriele d’Annunzio University of ChietiPescara, 66100 Chieti, Italy

In 2007, I was (LV) attending to a one-month period of my pediatric residency at the Children’s Hospital in New Orleans. The enormous destruction and significant loss of life caused by Katrina, an extremely powerful hurricane two years earlier, was still obvious everywhere. Katrina was the costliest hurricane to have hit the United States, surpassing the record of Hurricane Andrew in 1992 [1]. 

Similarly, the hurricane of SARS-CoV2 and the related COVID-19 interstitial pneumonia struck at the end of 2019, affecting more than 380,000,000 people and accounting for more than 5,700,000 deaths worldwide in the latest three years; many more victims than those claimed by the Vietnam War [2].

At the beginning of 2020, the first COVID-19 wave abruptly overwhelmed healthcare systems’ capacities with an acute saturation of intensive care unit beds [3]. As a consequence, the first challenge was triage for patients with severe acute respiratory failure [4]. 

The idea to “treat the ones who will benefit the most”, in some cases, led to patients not receiving the highest level of care. Clinical survival probability scores were highly advocated to optimize available local resources. One of these, 4C, performed well, and it has been validated in a large cohort of COVID-19 patients [5]. This score, considering age and sex of patient along with chronic conditions, respiratory rate, oxygen saturation, consciousness, urea, and C-reactive protein, has a good prediction of 30-day in-hospital mortality (AUC = 0.793) in younger and older patients.

At that time, a lockdown was the only way to limit the virus’ spread and overcome the growth of the epidemic wave, so many governments were forced to declare it [6]. Demand for noninvasive and invasive ventilatory support increased everywhere, especially in developing countries, leading to the collapse of many hospitals [7]. 

In response, healthcare systems rearranged their structures to compensate for this emergency (the REsponse phase), and anesthesiologists left operating theaters to help their colleagues in ICUs [8]. Critical care physicians also worked intensively outside their ICUs, supporting high-dependency units or other wards where personnel were rapidly trained to offer respiratory support to patients with moderate-to-severe pneumonia. High-flow nasal cannula (HFNC) and noninvasive ventilation were widely adopted by non-experts, including in resource-constrained settings, to treat a large cohort of patients outside the ICU [9]. 

Results have been promising with less severely ill patients who have benefited from HFNC use [10]. Consequently, some international experts in acute respiratory failure treatment have proposed broadening the adult respiratory distress syndrome (ARDS) Berlin definition to include patients on HFNC [11]. 

In COVID-19 interstitial pneumonia, a computed tomography (CT) scan shows devastating lung damage that is further confirmed by autoptic studies. However, the need to avoid viral spread throughout hospitals, and the severity of the disease that sometimes hinders the possibility of transporting these patients, have increased the use of ultrasound as a comprehensive head-to-toe bedside monitoring tool [12,13].

Use of ultrasound increased the suspicion of pulmonary embolism whenever an “enlarged right ventricle” was observed, which was confirmed by autoptic studies, demonstrating that patients with COVID-19 presented a high prevalence of pulmonary embolisms [14]. These findings changed the standard DVT prophylaxis, and critical-care physicians often moved toward a more aggressive anticoagulant treatment.

Some researcher put attention to the fact that COVID-related ARDS (C-ARDS) could sometime be different from classical ARDS because lung compliance could be higher in the former, despite severe hypoxemia [15]. A ventilator approach complying with the principles of “lung protection” and of personalized ventilation was, in any case, the foundation of respiratory support, both noninvasive and invasive, in C-ARDS patients, as for any other form of ARDS. Refractory COVID-19 hypoxemia was sometimes treated with extracorporeal oxygenation techniques in some centers, but available resources were the main limitation.

With the advent of the vaccine in 2021 and a better understanding of COVID-19 disease, succeeding epidemic waves have been characterized by a reduced wave height. However, healthcare systems had not yet returned to pre-COVID-19 situations [16]. 

Young anesthesiologists have seen a reduction in operating room availability since the early months of 2020, when many healthcare resources were diverted from surgical units and ambulatory settings to COVID-19 areas. At the peak of contagions, operating theaters were closed for any type of surgery, except for emergencies [17].

Nothing similar had happened in the last 50 years in Italy. Moreover, outpatient and day hospital medicine declined, causing patients affected by oncologic and chronic disease to experience difficulty in accessing hospital care [18].

This is the second challenge of the COVID-hurricane: to restore hospital activities other than COVID care to levels comparable to the pre-COVID era. Indeed, to recover and restore waiting lists, diagnostic-therapeutic activities should be increased (REstoration phase), taking into consideration that a cohabitation with endemic COVID will likely go on for some time.

It is difficult to predict how much time will be needed to fill this gap. If we compare COVID-19, not only to Katrina, but also to other disasters in history, such as the earthquake in Friuli-Venezia-Giulia, Italy, in 1976, it is legitimate to think that it will take some time to restore healthcare systems and make them ready to face future possible pandemics [19].

In fact, when hurricanes pass, the destruction remains, and the need for restoration and reengineering to ameliorate previous systems will take significant energy and time [20].

Many factors, including economic and social ones, will slow down (Scenario A in Figure 1) or speed up this reengineering (Scenario B in Figure 1).

In the figure, the level of routine acitivity (red line) and COVID-19-related activity (blue line) are described over time. During the emergency phase, with rapidly increasing hospital admissions (REsponse phase), routine activity rapidly declined to divert resources to COVID-related activity. During this phase, high COVID-19-related mortality was recorded. In the meantime, chronic disease patients and cancer surgeries received less resources, producing lists of overdue patients. As the pandemic declines, routine activity should be restored to eliminate the backlog. Due to cure delays that occurred in REsponse phase, during the REstoration phase an increased mortality, not directly related to COVID-19 infection, is observed. Meanwhile, long COVID syndromes may increase the number of disabled patients who will likely need additional care. It is difficult to predict how long the restoration phase will last, but taking into consideration other disaster examples, such as Katrina, it is reasonable to think the REstoration phase will take many years. Finally, healthcare systems will be reenginereed (the REengineering phase), with telemedicine probably being widely used to optimize human resources. Whether REengineering will be slower (Curve A) or faster (Curve B) will depend on the ability of national systems to direct flows of resources in an optimal manner. However, the most important issue is that healthcare system managers should draw up a priority list that takes the healthcare needs of citizens into account. In doing so, political counterparts should also direct funds in a targeted, rapid, and precise manner to improve healthcare activities, ideally to overcome any unforeseen circumstances (REengineering phase) [21]. 

Telemedicine will probably help in achieving this, and it should be an important part of this “Re-engineering process” also to strengthen “territorial” care (homecare and community hospitals) by supporting hospital care [22].

The role of family medicine will be fundamental in the coming years. General practitioners should be considered fundamental to establish priorities among patients, deciding who will be the first to access a hospital, and who can wait because of a stable chronic disease.

Programs of population screening for cancer, continuous treatments for chronic diseases, and the implementation of telemedicine will have to go through general practitioners because they are the ones that know the needs of people while also giving feedback to healthcare policy makers. In this way, we will probably experience a fast restoration period while optimizing available resources.

There will always be hurricanes, perhaps less frequent, but unfortunately still of extreme intensity. RE-RE-RE will not prevent them, but it will perhaps mitigate the damage and speed up the recovery. 

The COVID-19 hurricane has taught us that resources for healthcare systems should be calculated on the probability of extraordinary events, and not just on routine activities. Politician and economic advisors, however, usually tend to keep resources to “minimum”. As a consequence, the pendulum swings between sparing resources and increasing them for possible future extraordinary events.

The REengineering phase should address the issue of calculating the new number of needed healthcare professionals and facilities to face routine and extra routine events, otherwise, we will continue to chase the problem without remedying it.

## Figures and Tables

**Figure 1:**
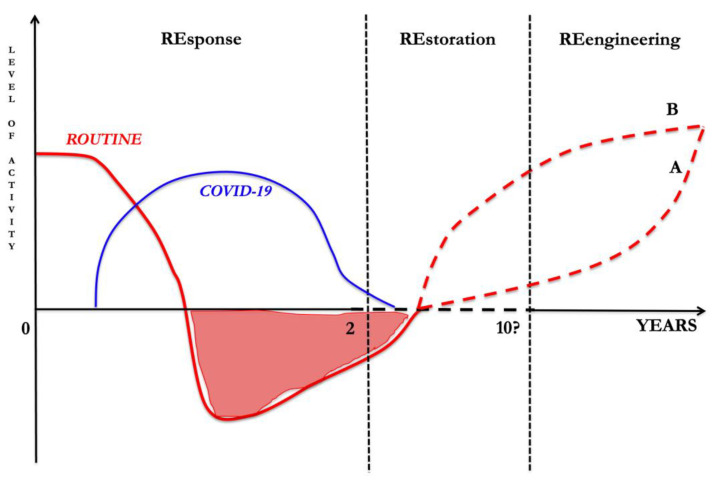
The RE-RE-RE approach to the pandemic.

## Data Availability

Not applicable.
